# Concordance of Chronic Conditions in Older Mexican American Couples

**Published:** 2005-06-15

**Authors:** Jim P Stimpson, M. Kristen Peek

**Affiliations:** Sealy Center on Aging, University of Texas Medical Branch; Department of Preventive Medicine and Community Health, University of Texas Medical Branch, Galveston, Tex

## Abstract

**Introduction:**

There is substantial evidence that marriage is beneficial to health, but evidence on whether the health status of one spouse is similar, or concordant, with the other spouse is limited. This study assessed whether a chronic condition of one spouse is a risk factor for the same chronic condition in the other spouse.

**Methods:**

The study used baseline data from the Hispanic Established Populations for the Epidemiologic Studies of the Elderly on 553 couples (1106 individuals) who are representative of approximately 500,000 older (≥65 years) Mexican Americans living in the southwestern United States. Logistic regression was used to predict six chronic conditions among couples: heart condition, stroke, hypertension, diabetes, arthritis, and cancer. Analyses were adjusted for age, education, U.S. nativity, blood pressure, body mass index, smoking, and alcohol consumption.

**Results:**

The wife's history of hypertension, diabetes, arthritis, and cancer was associated with higher odds that the husband would have these conditions. A history of hypertension, arthritis, and cancer in the husband was associated with higher odds that the wife would have these conditions.

**Conclusion:**

These results provide preliminary evidence that chronic conditions in one spouse are associated with an increased risk of developing like conditions in the other spouse among older Mexican American couples. We propose that the reciprocal influence that marital partners have on each other may be caused by shared living arrangements and shared health risks. Health promotion activities should target family systems. In particular, health providers should gather health histories not only from patients and their genetic family members but also from spouses.

## Introduction

As the number of older adults continues to rise and mortality rates decrease, health providers must treat a growing population of individuals with chronic illness. An important determinant of chronic illness is social context, and new studies are being designed to examine how a person's social environment shapes well-being (1,2). For example, evidence on the effect of neighborhoods is growing, but an equally important, if not more important, social context is marriage: marriage provides the primary source of social support and economic stability for married individuals, and this support and stability have been shown to be associated with physical health (3). The health benefits of marriage are well established (4). Marriage is associated with lower mortality rates (5,6), better physical health (7), and better psychological well-being (8). The positive benefit of marriage is particularly important for older couples as they cope with chronic illness (9,10).

A new line of research has emerged that examines the similarity, or concordance, of health between spouses. Concordance speaks directly to the social contextual forces that shape health. By sharing a living environment, resources, life events, and habits, individuals ultimately share health risks that may translate into having a disease they might otherwise not have in an alternative social context. Recent investigations of spousal concordance have used wives' well-being to predict husbands' well-being while controlling for wives' and husbands' physical health and vice versa. Most of the research on concordance has examined mental health or self-rated physical health (11-16). To the authors' knowledge, only two studies have used an objective measure of physical health. Using blood pressure as their outcome measure, these studies found that spouses' blood pressure was similar and that this similarity presented another risk factor for hypertension (17,18).

Most research on marriage and health has focused on white couples, with very little research among minority populations. This lack of research may represent a critical omission because couples among minority populations tend to have a high prevalence of certain types of diseases and disabilities (19). Moreover, the number of elderly Mexican Americans is growing, and health providers need more information on the unique needs of this group. This study examines older Mexican American couples residing in the southwestern United States to determine whether chronic conditions such as heart attack, stroke, hypertension, diabetes, arthritis, and cancer of one spouse increase the likelihood that the other spouse has these same conditions.

## Methods

### Sample

We performed secondary analysis using data from the Hispanic Established Populations for the Epidemiologic Studies of the Elderly, 1993–1994 (Hispanic EPESE) (20). The Hispanic EPESE collected baseline data from September 1993 through June 1994 on a sample of Mexican American adults aged 65 years and older who resided in five southwestern states: Arizona, California, Colorado, New Mexico, and Texas. The study used a multistage area probability sampling design. In the first stage, counties were selected if at least 60% of the county population consisted of older Mexican Americans. Within these, 300 census tracts were randomly chosen. From these 300 census tracts, three census blocks were randomly chosen. To obtain at least 400 households within each sampling unit, one to two additional census blocks were added. In the final stage of the Hispanic EPESE, in-person interviews were conducted with members of households who were aged 65 years and older and of Mexican origin; up to four members of each household were interviewed. Approximately 85% of Mexican American older adults live in the five states sampled; the Hispanic EPESE sampling design ensured that a sample representing approximately 500,000 older adults was obtained.  

The final data set of the Hispanic EPESE included data on 3050 respondents. Respondents ranged in age from 65 to 99 years and had attended school for an average of 5 years; 57% were female, and 47% had been born in Mexico (20). Because multiple members in each household aged 65 years and older were interviewed as part of the study, the Hispanic EPESE was able to gather data on both husbands and wives in approximately 30% of households, resulting in data on 553 couples (1106 individuals).

### Measures

Participants in the Hispanic EPESE were asked if a doctor had ever told them they had one of six chronic conditions in their lifetime: heart condition or attack, stroke, hypertension, diabetes, arthritis, or cancer (1 = reported condition, 0 = did not report condition). Three indicators of demographic characteristics were included in the models: age, education, and U.S. nativity. For the subsample of 553 couples, age was measured as continuous in years and ranged from 65 to 94. Education was a continuous measure in years and ranged from 0 to 17. A binary question asked respondents to identify if they had been born in the United States. Risk factors for many of the conditions were included in the models. Respondents reported if they currently smoked or drank alcohol. Height and weight for each respondent was recorded and converted into a body mass index (BMI) score when a score was 30 kg/m^2^ or higher. Blood pressure was measured while respondents were in a sitting position, with high blood pressure indicated by a value of 140 mm Hg or higher for the systolic measurement and 90 mm Hg or higher for the diastolic measurement.

### Data analysis

This study conceptualizes concordance as how the health of one spouse affects the health of the other spouse, using matched data from spouses. Logistic regression models were used to assess the associations between the chronic conditions of one spouse while controlling for the other spouse's covariates. The models were adjusted first for age, education, and U.S. nativity and then for smoking, alcohol consumption, high BMI, and high blood pressure. Therefore, if the husband's chronic condition predicts the similar condition for the wife when accounting for wives' risk factors, then this suggests evidence for concordance of chronic illness among these older Mexican American couples. All analyses were performed using SPSS software, version 12 (SPSS Inc, Chicago, Ill).

## Results

Table 1 shows the characteristics of our subsample of couples, which is comparable to Mexican Americans in the overall Hispanic EPESE sample (20) and presents preliminary evidence for concordance of some chronic conditions for older Mexican American couples. Husbands were slightly older than wives, but they had similar levels of education and were about as likely to have been born in the United States. Husbands were much more likely to smoke and drink alcohol than wives but had a lower incidence of high BMI. Finally, blood pressure was nearly identical, and this striking similarity extends to four of the six chronic conditions. Husbands reported slightly higher prevalence of stroke, diabetes, and cancer, but the largest gap was 3%. The prevalence of heart conditions was only 6% higher for husbands. However, this trend toward nearly identical prevalences with husbands slightly more likely to experience chronic conditions is different for the other two health conditions. Wives were more likely to have been diagnosed with hypertension and arthritis: the gap is 11% for hypertension and 16% for arthritis. We performed a *t* test to assess if the differences were significantly different and found all characteristics to be significantly different except diabetes, cancer, blood pressure, and education.

Table 2 presents the results from the logistic regression using the chronic conditions of the wife to predict the corresponding chronic conditions for the husband. The results are adjusted in two models; the first model is adjusted for age, education, and U.S. nativity of the husband, and the second is adjusted for age, education, U.S. nativity, blood pressure, body mass index, smoking, and alcohol consumption of the husband. All of the chronic conditions except stroke are significantly associated with an increased risk of the corresponding chronic condition for the husband in the first model. For example, the strongest effect found was for cancer; the husband's risk of cancer was 4.49 times more likely when his wife had been diagnosed with cancer. After adjusting for the husband's health risk factors, heart condition was not statistically significant, but hypertension, diabetes, arthritis, and cancer remained statistically significant. Having a wife with hypertension or diabetes increased the husband's likelihood of having hypertension or diabetes by about 75%; arthritis increased the odds for the husband by nearly 200% and cancer by more than 300%.

Table 3 presents the results from the logistic regression using the chronic conditions of the husband to predict the corresponding chronic conditions for the wife. Nearly the same results were found for the wife when adjusting for the wife's age, education, and U.S. nativity. Stroke was not statistically significant, but the wife's risk of developing a heart condition, hypertension, diabetes, arthritis, and cancer were positively associated with the husband's corresponding chronic conditions. After adjusting for the wife's health risk factors, hypertension, arthritis, and cancer remained significant predictors, but heart condition and diabetes were no longer statistically significant. Hypertension in the husband increased the odds for the wife of having hypertension by 63%; arthritis increased the odds by about 200% and cancer by more than 300%. These high odds, adjusted for relevant risk factors, suggest concordance among couples for certain chronic conditions.

## Discussion

The purpose of the research was to determine if the chronic condition of one spouse presented a risk factor for the other spouse for the same condition. In other words, if the husband has been diagnosed with diabetes, is the wife at greater risk for diabetes when controlling for relevant risk factors? Little research had been conducted on the concordance of health among spouses, and the research that does exist has largely focused on depression. Findings from the current study provide evidence that some chronic conditions are concordant among older Mexican American spouses even after adjusting for age, education, U.S. nativity, blood pressure, BMI, smoking, and alcohol consumption. Of the six conditions tested, only stroke was not statistically significant for both husbands and wives. The husband's risks for being diagnosed with hypertension, diabetes, arthritis, and cancer were significantly increased when the wife was diagnosed with the corresponding condition. The wife's risks for being diagnosed with hypertension, arthritis, and cancer were significantly increased when the husband was diagnosed with the corresponding condition. These results are consistent with the two other studies that used objective physical health measures, rather than mental health, to study concordance among couples (17,18). This pattern suggests that, along with individual risk factors, marital partners are an important influence on health, especially among older couples.

One of the strengths of this study is its focus on a group that has received very little attention — Mexican Americans. Based on a large, community-dwelling probability sample of older Mexican Americans, the findings provide more information about a group that is rapidly becoming a significant proportion of the elderly population. Older Mexican American spouses face a similar risk for low quality of life as white couples because of their declining health coupled with a diminished social support network (10). Older minority couples are an especially important population because they have high prevalence of certain diseases. For example, approximately 42% of the Mexican Americans in the Hispanic EPESE sample had self-reported hypertension in 1993. These data show that having a spouse with hypertension increases the odds of having hypertension by up to 75%.

One of the limitations of this research was the lack of other health behavior variables (e.g., diet, exercise) that are risk factors for major chronic diseases. It would be helpful to adjust the findings with these additional health behaviors, but another study that examined concordance among couples did not find evidence that concordance of health behaviors was related to concordance of blood pressure (18). Measures of quality and length of the marital relationship are also missing from the analysis. Previous research suggests that marital quality is an important factor to consider in studying marriage and well-being (6,21), but data on marital quality are not available in the Hispanic EPESE. Because we used data from matched pairs of spouses, length of marriage was controlled by default and therefore not included in the analysis. However, we did test models that included length of marriage, and the results did not change substantively. We included blood pressure as a covariate in the logistic models for hypertension because respondents could have been treated for high blood pressure prior to being interviewed, and excluding blood pressure from the analysis did not alter the coefficients. Finally, these data do not provide information on dates of diagnosis for medical conditions. Diagnosis of a condition in one spouse may increase the likelihood that the other spouse will be screened for the same condition, but the data only indicate whether the spouse has ever received a diagnosis of a medical condition by a doctor. Without dates of diagnosis, it is difficult to determine the effect of marriage on screening behavior.

Future research should identify the mechanisms and processes through which concordance operates. Some scholars have speculated that concordance results from assortative mating, which is the idea that healthy individuals select each other for marriage based on like or unlike characteristics (5,22). Presumably, healthier individuals are more likely to be married because they are able to find partners and maintain relationships. However, most people are healthy when they marry, and a longitudinal study concluded that concordance more likely results from shared environmental stressors and risk factors rather than assortative mating (23). Clearly, spouses share a common social context that could influence concordance, but differences in medical conditions and health behaviors persist between sexes. Potential avenues for future research should use longitudinal data to explore how quality of the marital relationship affects concordance, identify the physiological and psychological processes of how a chronic condition in one spouse affects the other spouse, and examine a wider range of health outcome measures.

Although more research is needed, the findings from the current study have clinical and policy implications. Having a spouse with a chronic condition may increase the odds of developing or worsening a similar condition because of shared life circumstances and environment. Marital partners have a mutual influence on each other. For example, one partner may eat well, exercise, not smoke, and drink in moderation, but if the other spouse does not share those lifestyle characteristics, particularly if the other spouse smokes, then the unhealthy behaviors may have an effect on the healthier spouse. Likewise, the positive health behaviors of one spouse may encourage the less healthy spouse to adopt positive health behaviors. Perhaps the best example is diet: marriage partners share a kitchen and the financial resources to buy food, so it is likely that couples eat similar meals, at least at home. One spouse's food preference may dominate the meal decisions, causing one partner to concede to the other (either to buy healthy food or less healthy food). In this way, one's risk for developing a chronic condition may be partly due to the health behavior of the spouse. Therefore, this study suggests that health promotion and disease prevention should target the family system. Several studies have noted that health care providers pay little attention to family members and instead focus interventions on the patient (24,25). Because characteristics of marital and family relationships can influence disease progression and management, health providers should gather a health history of the spouse in addition to the health histories of genetically related family members.

## Figures and Tables

**Table 1:** Characteristics of Participants in Study on Mexican American Couples Aged 65 Years and Older (n = 553 couples)^a^

** Characteristic**	**Husband**	**Wife**
Age, mean years (SD)	73.9 (6.3)	70.9 (5.2)
Education, mean years (SD)	5.2 (3.9)	5.2 (3.9)
U.S. nativity	58	62
Systolic blood pressure ≥140 mm Hg	31	33
Diastolic blood pressure ≥90 mm Hg	21	20
BMI ≥30 kg/m^2^	21	37
Current smoker	15	7
Current alcohol consumer	20	4
Heart condition	13	7
Stroke	8	5
Hypertension	34	45
Diabetes	25	24
Arthritis	30	46
Cancer	6	5

a Source is Hispanic Established Populations for the Epidemiologic Studies of the Elderly, 1993–1994 (20). Numbers are percentages unless otherwise indicated.

**Table 2:** Husband’s Risk of Chronic Condition When Wife Is Diagnosed With the Condition, Mexican American Couples Aged 65 Years and Older^a^

	**Chronic Condition**					

	**Heart Condition OR (95% CI)**	**Stroke OR (95% CI)**	**Hypertension OR (95% CI)**	**Diabetes OR (95% CI)**	**Arthritis OR (95% CI)**	**Cancer OR (95% CI)**
**Model 1^b^**	2.40 (1.06-5.46)	1.33 (0.37-4.74)	1.75 (1.22-2.52)	1.77 (1.14-2.74)	2.80 (1.90-4.12)	4.49 (1.67-12.13)
**Model 2^c^**	2.23 (0.97-5.12)	1.09 (0.30-3.96)	1.75 (1.21-2.54)	1.78 (1.14-2.79)	2.83 (1.92-4.18)	4.47 (1.61-12.42)

a Data source is Hispanic Established Populations for the Epidemiologic Studies of the Elderly, 1993–1994 (20). OR indicates odds ratio; CI, confidence interval.

b Adjusted for age, education, and U.S. nativity of the husband.

c Adjusted for age, education, U.S. nativity, blood pressure, body mass index, smoking, and alcohol consumption of the husband.

**Table 3:** Wife’s Risk of Chronic Condition When Husband Is Diagnosed With the Condition, Mexican American Couples Aged 65 Years and Older^a^

** **	**Chronic Condition**					

	**Heart Condition OR (95% CI)**	**Stroke OR (95% CI)**	**Hypertension OR (95% CI)**	**Diabetes OR (95% CI)**	**Arthritis OR (95% CI)**	**Cancer OR (95% CI)**
**Model 1^b^**	2.32 (1.04-5.17)	1.21 (0.34-4.24)	1.66 (1.16-2.39)	1.64 (1.07-2.54)	2.90 (1.97-4.28)	4.78 (1.76-12.97)
**Model 2^c^**	2.24 (0.99-5.05)	1.07 (0.30-3.80)	1.63 (1.13-2.36)	1.53 (0.98-2.39)	2.96 (1.98-4.42)	4.65 (1.61-13.46)

a Data source is Hispanic Established Populations for the Epidemiologic Studies of the Elderly, 1993–1994 (20). OR indicates odds ratio; CI, confidence interval.

b Adjusted for age, education, and U.S. nativity of the wife.

c Adjusted for age, education, U.S. nativity, blood pressure, body mass index, smoking, and alcohol consumption of the wife.
